# *Enterocytozoon bieneusi* in fecal samples from calves and cows in Austria

**DOI:** 10.1007/s00436-022-07733-y

**Published:** 2022-11-17

**Authors:** Katharina Lichtmannsperger, Josef Harl, Sarah Rosa Roehl, Julia Schoiswohl, Cassandra Eibl, Thomas Wittek, Barbara Hinney, Sandra Wiedermann, Anja Joachim

**Affiliations:** 1 Department for Farm Animals and Veterinary Public Health, University Clinic for Ruminants, University of Veterinary Medicine Vienna, Vienna, Austria; 2 Institute of Pathology, Department of Pathobiology, University of Veterinary Medicine Vienna, Vienna, Austria; 3 Institute of Parasitology, Department of Pathobiology, University of Veterinary Medicine Vienna, Vienna, Austria

**Keywords:** Antimicrobial treatment, Cattle, Genotyping, Microsporidia, ITS, Zoonosis

## Abstract

*Enterocytozoon bieneusi* is an obligate intracellular pathogen that infects livestock, companion animals, and wildlife and has the potential to cause severe diarrhea especially in immunocompromised humans. In the underlying study, fecal samples from 177 calves with diarrhea and 174 adult cows originating from 70 and 18 farms, respectively, in Austria were examined for the presence of *E. bieneusi* by polymerase chain reaction targeting the Internal Transcribed Spacer 1 (ITS1) region. All positive samples were further sequenced for genotype determination. Overall, sixteen of the 351 (4.6%) samples were positive for *E. bieneusi*, two of the 174 samples from cows (1.2%) and 14 of the 177 samples from calves (7.9%). In total, four genotypes, J (*n* = 2), I (*n* = 12), BEB4 (*n* = 3), and BEB8 (*n* = 1), were identified. The uncorrected *p*-distance between the four ITS1 lineages (344 bp) ranges from 0.3% to 2.9%. The lineages differ by 1 bp (I and J), 2 bp (J and BEB4), and 3 bp (I and BEB4), respectively, and BEB8 differs by 7 to 10 bp from the latter three lineages. Two of the *E. bieneusi*-positive calves showed an infection with two different genotypes. *E. bieneusi* occurred significantly more often in calves > 3 weeks (8/59) than in calves ≤ 3 weeks (6/118), respectively (*p* = 0.049). Calves with a known history of antimicrobial treatment (50 of 177 calves) shed *E. bieneusi* significantly more often than untreated calves (*p* = 0.012). There was no statistically significant difference in *E. bieneusi* shedding in calves with or without a medical history of antiparasitic treatment (*p* = 0.881). Calves showing a co-infection with *Eimeria* spp. shed *E. bieneusi* significantly more often than uninfected calves (*p* = 0.003). To our knowledge, this is the first report of *E. bieneusi* in cattle in Austria. Cattle should be considered as a reservoir for human infection since potentially zoonotic *E. bieneusi* genotypes were detected.

## Introduction

Microsporidia are obligate intracellular pathogens that infect a wide range of invertebrates and vertebrates and have the potential to infect humans. This taxon contains at least 1700 species in approximately 220 genera, of which ten genera (17 species) have been described in humans (Han et al. 2021). The two taxa that frequently infect humans are *Enterocytozoon bieneusi* and *Encephalitozoon* spp., which have also been described in wild animals, domestic pets, and food-producing animals (Didier 2005, Hinney et al. 2016). In particular, immunocompromised humans, *e.g*., AIDS patients or organ transplant recipients, are at higher risk for infection (Han et al. 2021). *Enterocytozoon bieneusi* is the microsporidian species that most frequently causes human infection and is responsible for 90% of cases of chronic diarrhea in AIDS patients (Weber et al. 1994; Sak et al. 2011).

However, *E. bieneusi* can also lead to asymptomatic and symptomatic infections in immunocompetent humans (Didier and Weiss 2011; Sak et al. 2011). The main transmission route is oral ingestion of food and/or water contaminated with spores (Mathis et al. 2005). Since microsporidian spores are very small (0.7 to 1.0 μm by 1.1 to 1.6. μm), detection in samples by light microscopy is difficult and requires staining. For the identification of. *E. bieneusi* to the genus or species level, molecular methods have the highest sensitivity and specificity (Franzen and Müller 1999; Zhang et al. 2021). Among the molecular detection methods, amplification and sequencing based on the Internal Transcribed Spacer 1 (ITS1) region was established as a reliable tool to identify species and genotypes of Microsporidia (Dong et al. 2010). Molecular genetics also allow differentiation between host-specific and zoonotic *E. bieneusi* genotypes. Genotypes can be divided into separate groups according to host specificity. Groups 1 and 2 have been detected in multiple species including humans and are considered to be zoonotic; groups 3 to 11 seem to be more specific to animals (Li et al. 2019). Nevertheless, suggested ruminant-specific genotypes (J, I, BEB4, and BEB6) have also been reported in humans (Jiang et al. 2015; Yu et al. 2019). *Enterocytozoon bieneusi* was first reported in cattle by Rinder et al. (2000), who detected genotypes J and I in cattle in Germany. The ITS1 sequences of genotypes J and I from samples from cattle from Germany were identical to BEB1 and BEB2 originating from cattle in the USA, respectively (Rinder et al. 2000; Sulaiman et al. 2004). A recent meta-analysis showed that genotypes BEB4, J (BEB1), and I (BEB2) were the most frequently reported genotypes in cattle globally (Taghipour et al. 2022). Genotype BEB8 belongs to group 2 and has been described in bovines, non-human primates, and bats (Li et al. 2019).

To date, there is no information on the occurrence of *E. bieneusi* in cattle in Austria. The objective of the study was to describe the occurrence of *E. bieneusi* in fecal samples from diarrheic calves and clinically healthy cows in Austria, and we hypothesized that *E. bieneusi* frequently occurs in these samples.

## Materials and methods

### Animal selection and sample collection

#### Calves

During the years 2017 and 2018, veterinarians and farmers from all over Austria were asked to participate in the study. If the farmer was willing to participate, one of the first authors (KL) or the local veterinarian visited the farms and collected the fecal samples. Additionally, specific information on the included calves (medical history, clinical examination) was collected. Clinical examination was performed according to Baumgartner and Wittek (2018). The inclusion criteria for the calves were signs of diarrhea at the time of the herd visit (soft, liquid, or watery feces) and less than 180 days of age. At least 10 g or 10 ml of feces was collected during the farm visit using a urine collection cup. In total, 177 calves with diarrhea originating from 70 farms all over Austria were included. The fecal samples were immediately transported to the University Clinic for Ruminants Vienna by one of the first authors (KL). All samples were analyzed for the following calf diarrhea-causing pathogens: protozoa (*Cryptosporidium* spp., *Giardia intestinalis*, and *Eimeria* spp.), viruses (bovine Rotavirus A and bovine Coronavirus), and bacteria (*Clostridium perfringens*). The methods implemented for the detection of other enteropathogens (viruses, bacteria, and protozoa) have been described elsewhere (Lichtmannsperger et al. 2019, Lichtmannsperger et al. 2022). The remaining fecal and DNA samples were stored at minus 80 °C for further investigations.

#### Cows

In 2020, one hundred seventy-four cows from 18 conveniently selected dairy farms from the province of Salzburg, Austria, were included. All of the dairy farmers were customers of one local veterinary practice, and samples were taken on occasion of a farm visit for herd health checkup. During the farm visit, general information on farm characteristics and detailed information on the cows were gathered. All cows were clinically healthy. Fecal samples were collected using disposable gloves and transported to the University Clinic for Ruminants Vienna under cooling conditions within one day. Upon arrival, the samples were immediately frozen at minus 80 °C until further investigations.

### DNA extraction

All fecal samples were thawed at room temperature, transferred into a 20 ml collection cup, and homogenized using a wooden mouth spatula before approximately 200 mg aliquots were used for DNA extraction. Extraction was performed using the NucleoSpin® Soil kit (Macherey–Nagel GmbH & Co. KG, USA) according to the manufacturer’s specifications. In brief, lysis buffer (SL1) was used for sample preparation without lysis condition adjustment, and DNA elution was carried out using 100 μl of elution buffer. DNA was stored at minus 20 °C until used in PCR analysis.

### PCR for genotyping

*Enterocytozoon bieneusi* was screened based on the ITS1 region by nested PCR under the following conditions: the cycling protocol for both reactions included an initial cycle of 94 °C for 2 min, followed by 20 cycles (nest 1) or 35 cycles (nest 2) of 94 °C for 30 s, 57 °C for 30 s, 72 °C for 60 s, and a final extension of 72 °C for 10 min. The reaction volume was 25 μl containing 2 μl of genomic DNA template, standard PCR buffer (5xGreen GoTaq® Reaction Buffer, Promega USA), 10 mM of each dNTP, 1.25 U of Taq polymerase (GoTaq® G2 DNA Polymerase, Promega, USA), and 10 pmol of each oligonucleotide primer (Table 1).

**Table 1:** Primers utilized in nested PCR reactions amplifying the entire ITS1 region of *Enterocytozoon bieneusi* from fecal samples originating from diarrheic calves and healthy cows in Austria. The primers are located in the *18S* rRNA and *5.8S* rRNA gene sequences flanking the ITS1

PCR round	Primers	Primer sequence	Amplicon size (bp)
1	Ebien_ITS1_F1	for: CAGATGGTCATAGGGATGAAGAGC	455
	Ebien_ITS1_R	rev: CATTCCGTGAGGACTTTTCGC	
2	Ebien_ITS1_F2	for: CTTCGGCTCTGAATATCTATGGCT	389
	Ebien_ITS1_R	rev: CGACGGATCCAAGTGATCCTG	

### Sequencing of PCR products

The PCR products were sequenced in both directions at LGC Genomics GmbH (Berlin, Germany). The raw forward and reverse sequences (and electropherograms) were carefully checked and aligned with Bioedit v.7.0.8.0 (Hall 1999). Sequences were compared with reference sequences in GenBank® using the Basis Local Alignment Searching Tool (BLAST; https://blast.ncbi.nlm.nih.gov/Blast). The obtained sequences are deposited in GenBank® under the accession numbers OP455917-OP455934.

### Statistical analysis

Statistical analysis was performed using IBM® SPSS® Statistics Version 27 (IBM®, New York, USA) and Microsoft Excel 2010. To describe the differences in parasite occurrence between age groups, antimicrobial treatment, antiparasitic treatment, and the additional diseases, chi-square tests were applied. All tests were calculated with a significance level of *p* < 0.05.

## Results

### Calves

#### General information

In total, 177 calves with diarrhea originating from 70 farms were included. The animals were between one and 164 days old (median = 12; mean = 27). On average, 2.5 calves were sampled per farm (range = 1 to 10 calves). Female (108) and male (69) calves were included. The calves were Simmental (141), Holstein (8), Brown Swiss (1), or crossbreeds (27).

#### PCR results

In total, fourteen of the 177 included samples were positive for *E. bieneusi* by PCR. Eight of the 14 *E. bieneusi*-positive calves originated from dairy farms, four from fattening units, and two from cow-calf operations. The positive calves originated from 12 different farms; two farms had two positive calves each. The 14 positive calves were between 7 and 164 days of age (mean = 41.8 days, median = 29.5). *Enterocytozoon bieneusi* occurred significantly more often in calves > 3 weeks (8/59) than in calves ≤ 3 weeks (6/118) days, respectively (*p* = 0.049). Six of the positive animals were female (6/108) and eight were male (8/69) (*p* = 0.244). Nine of the calves were born on the farm and five were purchased at an auction or from calf dealers. The duration of diarrhea before the farm visit was for 1 to 3 days and > 3 days in ten and four positive calves, respectively. Eight animals had a very good and six animals had a reduced feed intake at the time of sample collection. The body condition was good in 13 calves; one calf was in poor body condition. The hydration status was normal in 12 of the investigated calves; two animals showed moderate dehydration.

#### Medical history of diarrheic calves

Based on the medical history, fifty diarrheic calves received antibiotics prior to sampling. Of the 50 treated animals, 31 (31/50) were less or equal than three weeks of age and 19 (19/50) were older than 3 weeks of age. There was no significant difference between calves receiving antibiotics ≤ 3 and > 3 weeks of age (*p* = 0.409). Table 2 gives an overview on the implemented antimicrobial classes. *Enterocytozoon bieneusi* infection status (PCR results) and the differences in medical history regarding the treatment with antimicrobial, antiprotozoal, and antiphlogistic drugs are shown in Table 3. Information on the *E. bieneusi* status (positive or negative) and concurrent infections with other calf diarrhea-causing pathogens is summarized in Table 4.

**Table 2:** Application of antimicrobial active substances used in neonatal calf diarrhea according to the medical history of the calves. In total, 50 of the 177 sampled calves had a medical history of antimicrobial treatment

Antimicrobial compound class	*N* calves
Sulfonamides	9
Tetracyclines	19
Penicillins	2
Quinolones*	5
Cephalosporins (4^th^ generation)*	4
Combination of multiple antimicrobial classes	2
No detailed information on antimicrobial class	9
*Total treated calves*	*50*

*Enrofloxacin and cefquinome are defined as the highest priority critically important antimicrobials by the World Health Organization

**Table 3:** Overview on the *Enterocytozoon bieneusi* infection status and the medical history of treatments or an additional disease. All tests were calculated with a significance level of *p* < 0.05

		*E. bieneusi* status			
		Positive	Negative	Total	*p* value
Antimicrobial treatment	Yes	8	42	50	0.012
	No	6	121	127	
	Total	14	163	177	
Antiprotozoal treatment*	Yes	2	21	23	0.881
	No	12	142	154	
	Total	14	163	177	
Antiphlogistic treatment	Yes	2	29	31	0.741
	No	12	134	146	
	Total	14	163	177	
Additional diseases^#^	Yes	2	22	24	0.934
	No	12	141	153	
	Total	14	163	177	

*Antiprotozoal treatment including halofuginone and toltrazuril/diclazuril; ^#^besides diarrhea: omphalitis and/or pneumonia

**Table 4:** Overview on the *Enterocytozoon bieneusi* infection status in the included calves and concurrent infections with other agents of calf diarrhea. All tests were calculated with a significance level of *p* < 0.05 (in bold)

Infectious agents		*E. bieneusi* status			
		Positive	Negative	Total	*p* value
*Giardia intestinalis*	Yes	5	43	48	0.451
	No	9	120	129	
	Total	14	163	177	
*Cryptosporidium* spp.	Yes	5	93	98	0.123
	No	9	70	79	
	Total	14	163	177	
*Eimeria* spp.	Yes	6	21	27	**0.003**
	No	8	42	150	
	Total	14	163	177	
*Clostridium perfringens*	Yes	3	50	53	0.469
	No	11	113	124	
	Total	14	163	177	
Bovine Rotavirus A	Yes	2	40	42	0.387
	No	12	123	135	
	Total	14	163	177	
Bovine Coronavirus	Yes	7	53	60	0.185
	No	7	110	117	
	Total	14	163	177	

### Cows

All fecal samples of cows (*n* = 174) originated from dairy farms (*n* = 18) from the province of Salzburg, Austria. On average, thirty-six dairy cows were housed on the dairy farms (median = 33.5, range = 13–70). The majority of animals were Simmental breed (83.3%) followed by Holstein (3.9%) and the remaining 12.8% of cows were Brown Swiss, Pinzgauer, and crossbreeds. Fecal samples from two cows showed a positive PCR result. The two positive Simmental cows originated from the same dairy farm. At the time of sample collection, all the dairy cows were lactating.

### Distribution and genetic characterization of *E. bieneusi* genotypes

**Fig. 1:**
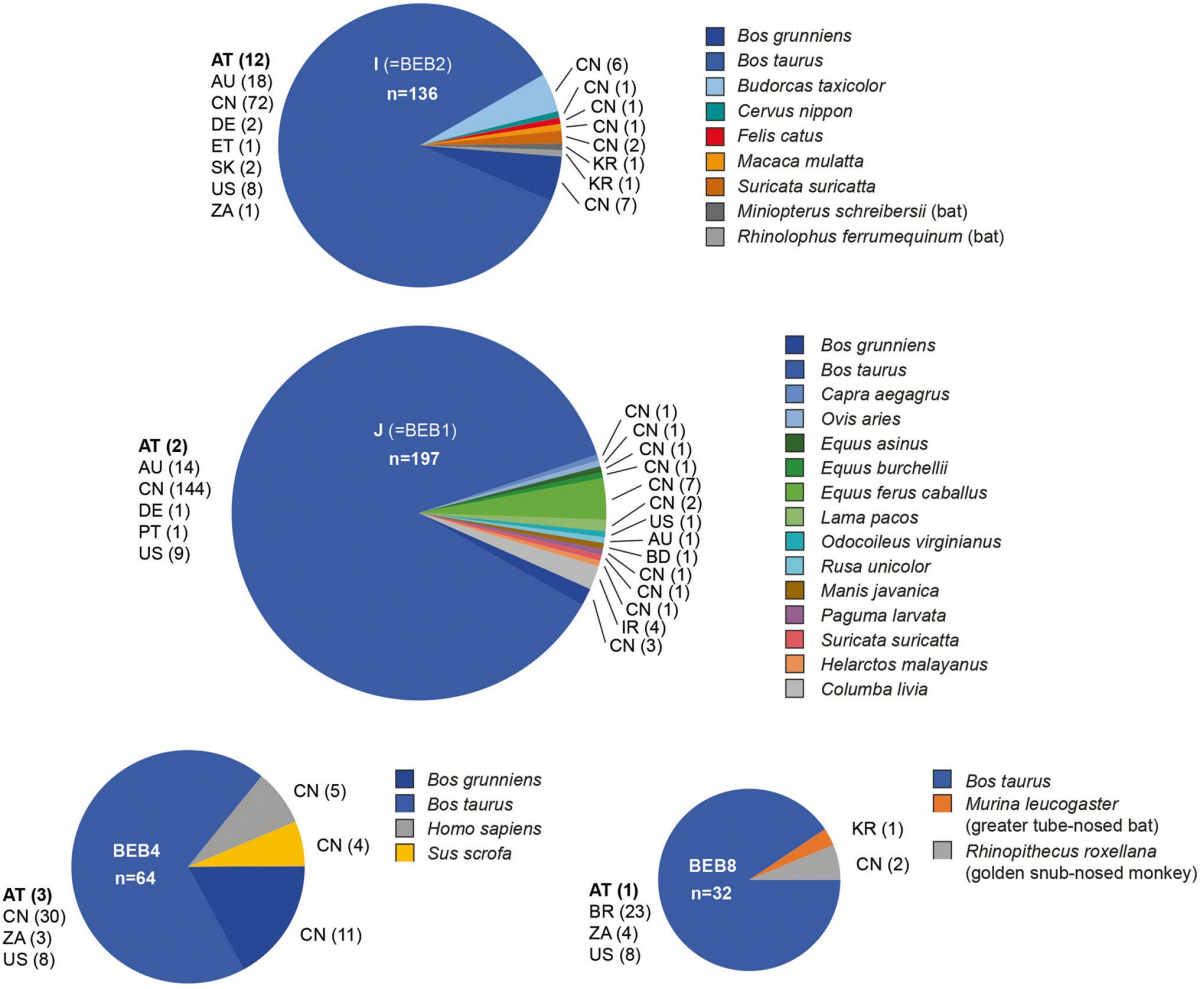
Pie charts showing the number of records per country and host species for the documented four *Enterocytozoon bieneusi* genotypes (I, J, BEB4, and BEB8). The country codes (ISO 3166–1) stand for Austria (AT), Australia (AU), Bangladesh (BD), Brazil (BR), China (CN), Egypt (ET), Germany (DE), Ireland (I) Portugal (PT), South Korea (KR), Slovakia (SK), United States of America (US), and Zaire (ZA). The numbers in brackets following the country codes refer to the number of records per animal species and country

The analysis of the ITS1 sequences revealed the presence of four genotypes (J, I, BEB4, and BEB8). The most prevalent genotype in the present study was I (66.7%, 12/18), followed by BEB4 (16.7%, 3/18), J (11.1%, 2/18), and BEB8 (5.6%, 1/18). Two genotype I-positive calves showed a concurrent infection with genotype J and BEB4. The two positive cows harbored genotype I. We downloaded all ITS1 sequences from NCBI GenBank and found 677 unique *E. bieneusi* lineages, of which 107 were isolated from humans and mostly also from various animal species. Most of these lineages are from unpublished studies, and the data is available on GenBank only. We therefore decided to only show the information on host species and country for the four ITS1 lineages found in the present study (Fig. 1). BEP4 is the only one of the four lineages which was isolated from five humans, all sampled in China.

## Discussion

This survey was part of a larger study on the occurrence of enteropathogens (*viruses, bacteria, and protozoa*) in calves with diarrhea in Austria (Lichtmannsperger et al. 2019, 2020, 2022). To our knowledge, this is the first report of *E. bieneusi* in calves and cows in Austria.

Recently, a meta-analysis has been published on the global prevalence of *E. bieneusi* in cattle. A pooled prevalence of cattle microsporidiosis of 13.9% has been described (Taghipour et al. 2022). The average *E. bieneusi* infection rates ranged from 2.0% to 21.4% (Zheng et al. 2020). The cumulative prevalence in the present investigation was 4.6%, which is comparatively low compared to these studies.

A higher prevalence has been described in pre-weaned calves (less than 6 months of age), female cattle, and cattle with diarrhea, but without statistical significance (Taghipour et al. 2022). In contrast to other studies, where it has been described that weaned calves had a higher prevalence than pre-weaned calves with 15.9% positive and 9.1% positive, respectively (*p* < 0.01) (Wang et al. 2019), in the present study, the occurrence of *E. bieneusi* in calves (14 of 177 calves positive) was 7.9% and in cows (2 of 174 cows positive) 1.1%, respectively (*p* = 0.005). *E. bieneusi* occurred significantly more often in calves > 3 weeks than in calves ≤ 3 weeks days, respectively (*p* = 0.049).

All calves were suffering from diarrhea and cows were healthy. The discrepant values among the studies might result from various factors such as differences in the animal management systems (*e.g*., loose housing, tethered housing, and outdoor and indoor housing), sample sizes (random selection or convenient sampling), climatic and environmental conditions, and the health status of the animals. For instance, the majority of available literature on the occurrence of *E. bieneusi* originate from Asia, especially China, and North America, the USA (Taghipour et al. 2022). Therefore, the comparison between these studies and the underlying investigation is limited.

The occurrence of *E. bieneusi* has been investigated in 314 pre-weaned diarrheic and healthy calves in Korea (Hwang et al. 2020). They found an overall *E. bieneusi* prevalence of 16.9% and a significantly higher prevalence in diarrheic calves aged from one to 20 days old. A longitudinal study carried out in China investigated 250 fecal samples from 25 calves with differing fecal consistency (formed, loose, and liquid feces) throughout a sampling period of ten weeks (Zhao et al. 2020). The highest infection rate was recorded at six weeks of age which is also in accordance to the present investigation where the mean age of the infected animals was 41.8 days (median = 29.5 days). Nevertheless, in both studies, *E. bieneusi* has also been detected in calves with normal fecal consistency (Hwang et al. 2020; Zhao et al. 2020). In previous investigations, no difference in *E. bieneusi* infection status (positive or negative) and *Cryptosporidium* spp. or *Giardia intestinalis* co-infections was detected (Hwang et al. 2020). In the present study, a significant difference has been found only between calves infected with *Eimeria* spp. The 27 *Eimeria* spp.-positive calves were on average 65.4 days old (range = 19 to 164, median = 56.0). Since only diarrheic animals were included, it cannot be concluded if there is a real difference in calves positive for *E. bieneusi* with/without *Eimeria* infection.

Calves with a medical history of antimicrobial treatment shed *E. bieneusi* significantly more often than the animals with no history of antimicrobial treatment. In total, fifty calves were treated with antibiotics and nine of these received the highest priority critically important antimicrobials (WHO 2019). There is an urgent need for more scientific data allowing to define the best practices for the treatment of neonatal calf diarrhea (Eibl et al. 2021). In healthy calves, it has been well known that the gut microbiota shows a higher richness, evenness, and diversity in comparison to diarrheic calves (Gomez et al. 2022). Additionally, the administration of antimicrobials (e.g., fluoroquinolones or penicillins) has been described to alter the fecal microbiota in calves (Beyi et al. 2021; Grønvold et al. 2011). The role of parasites and fungi in the gut microbiota of diarrheic calves and the combined use of antimicrobial treatment have to be further investigated.

It has been described that the *E. bieneusi* genotypes shed by young calves changed in the first ten weeks of life (Zhao et al. 2020); therefore, it might be possible that some genotypes might be underreported. The initially suggested cattle-specific genotype BEB4, detected in three samples from calves in Austria, is also found in humans (Zhang et al. 2011). In dairy cows, the suggested host-specific genotype I was detected. Genotype I has also been detected in multiple counties and species in China (*n* = 72) (Wang et al. 2016; Tao et al. 2020), Australia (18) (Zhang et al. 2019a), the USA (8) (Sulaiman et al. 2004), Germany (2) (Rinder et al. 2000), Slovakia (2) (Valenčáková and Danišová 2019), Ethiopia (1) (Wegayehu et al. 2020), and South Africa (1) (Samra et al. 2012). In China, genotype I was found in the yak *Bos grunniens* (7) (Wu et al. 2019), the takin *Budorcas taxicolor* (6) (Zhao et al. 2015), the sika deer *Cervus nippon* (1) (Tao et al. 2020), the cat *Felis catus* (1) (Karim et al. 2014a), the rhesus macaque *Macaca mulatta* (Karim et al. 2014b), and the meerkat *Suricata suricatta* (2) (Li et al. 2015).

Genotype J was isolated from cattle in Austria (2), China (144) (Wang et al. 2016), Australia (14) (Zhang et al. 2019b), Germany (1) (Rinder et al. 2000), Portugal (1) (Lobo et al. 2006), and the USA (9) (Sulaiman et al. 2004). In China, genotype J was also found in the horse *Equus ferus caballus* (7) (Li et al. 2020), the goat *Capra aegagrus* (1) (Shi et al. 2016), the donkey *Equus asinus* (1) (Yue et al. 2017), the palm civet *Paguma larvata* (1) (Yu et al. 2020), the zebra *Equus quagga* (1), the lama *Vicugna pacos* (2), the sheep *Ovis aries* (1), and the meerkat *Suricata suricatta* (1) (Li et al. 2015). The genotype was also isolated from the Malayan pangolin *Manis javanica* in Bangladesh (1) (Karim et al. 2020), the white-tailed deer *Odocoileus virginianus* in the USA (1) (Santin and Fayer 2015), and the sambar *Rusa unicolor* (1) in Australia (Zhang et al. 2018). Genotype BEB4 was isolated from cattle in Austria (3), China (30) (Tao et al. 2020), the USA (8) (Sulaiman et al. 2004), and South Africa (3) (Samra et al. 2012). Moreover, it was found in the yak *Bos grunniens* (11) (Wu et al. 2019), the pig *Sus scrofa* (4), and humans in China (5) (Zhang et al. 2011). Genotype BEB8 was isolated from cattle in Austria (1), Brazil (23) (da Silva Fiuza et al. 2016), South Africa (4) (Samra et al. 2012), and Ethiopia (1) (Wegayehu et al., unpublished; accession no. MT231510). The genotype was also found in the greater tube-nosed bat *Murina leucogaster* (1) in South Korea (Lee et al. 2018) and the golden snub-nosed monkey *Rhinopithecus roxellana* (2) in China (Yu et al. 2017).

Since zoonotic genotypes of *E. bieneusi* have been detected in cattle, they need to be recognized as reservoirs of zoonotic *E. bieneusi* in Austria. Since the detection of microsporidia is not part of the routine water analysis nor the routine diarrhea-diagnostic panel in humans, it is unknown to what extent the pathogen is present in the environment and the population in Austria.

One of the limitations of our study was that we performed convenient and not randomized sampling and included only diarrheic calves and not healthy ones and also no other age groups, so the true prevalence of *E. bieneusi* in Austrian cattle is still unknown. The intermittent shedding of *E. bieneusi* needs to be taken into account since the cross-sectional study design with single individual samples might still underreport the prevalence and the range of genotypes found.

In conclusion, the present study is the first report of *E. bieneusi* in diarrheic calves and healthy cows from Austria. Fourteen (7.9%) of the 177 calves and two (1.1%) of the 174 included cows were positive for *E. bieneusi*, respectively. The observed occurrence of potential zoonotic genotypes emphasizes the possible role of cattle in the transmission of *E. bieneusi* to humans in Austria.

## Data Availability

The obtained sequences are deposited in GenBank® under the accession numbers OP455917-OP455934.

## Declarations

### Disclaimer

The sponsor did not participate in the study planning, data analysis, and manuscript compilation.
